# Molecular genetic investigation of sporadic renal cell carcinoma: analysis of allele loss on chromosomes 3p, 5q, 11p, 17 and 22.

**DOI:** 10.1038/bjc.1994.44

**Published:** 1994-02

**Authors:** K. Foster, P. A. Crossey, P. Cairns, J. W. Hetherington, F. M. Richards, M. H. Jones, E. Bentley, N. A. Affara, M. A. Ferguson-Smith, E. R. Maher

**Affiliations:** Department of Pathology, Cambridge University, UK.

## Abstract

**Images:**


Br. J. Cancer (1994), 69, 230-234 © Macmillan Press Ltd., 1994

### Molecular genetic investigation of sporadic renal cell carcinoma: analysis of allele loss on chromosomes 3p, 5q, 11p, 17 and 22

K. Foster[1], P.A. Crossey[1], P. Cairns[2], J.W. Hetherington[3], F.M. Richards[1], M.H. Jones[4], E. Bentley[1], N.A. Affara[1], M.A. Ferguson-Smith[1] & E.R. Maher[1]

[1]Department of Pathology, Cambridge University, Cambridge, UK; [2]Marie Curie Research Institute, Oxted, UK; [3]Department of Urology, The Princess Royal Hospital, Hull, UK; [4]Cancer Institute, Tokyo, Japan.

**Summary** To investigate the role of tumour-suppressor genes on the short arm of chromosome 3 in the mechanism of tumorigenesis in non-familial renal cell carcinoma, we analysed 55 paired blood-tumour DNA samples for allele loss on chromosome 3p and in the region of known or putative tumour-suppressor genes on chromosomes 5, 11, 17 and 22. Sixty-four per cent (35/55) of informative tumours showed loss of heterozygosity (LOH) of at least one locus on the short arm of chromosome 3, compared with only 13% at the p53 tumour-suppressor gene and 6% at 17q21. LOH at chromsome 5q21 and 22q was uncommon (2-3%). Detailed analysis of the regions of LOH on chromsome 3p suggested that, in addition to the VHL gene in chromosome 3p25-p26, mutations in one or more tumour-suppressor genes in chromosome 3p13-p24 may be involved in the pathogenesis of sporadic renal cell carcinoma (RCC). We also confirmed previous suggestions that chromosome 3p allele loss is not a feature of papillary RCC ($P<0.05$).

Renal cell carcinoma (RCC) is an important human cancer whose aetiology is poorly understood. A small proportion of cases (approximately 2%) occur in patients with an inherited predisposition to RCC (Maher & Yates, 1991). The most common hereditary form of RCC is von Hippel-Lindau (VHL) disease, a dominantly inherited familial cancer syndrome predisposing to retinal and central nervous system haemangioblastomas, RCC and phaeochromocytoma (Maher *et al.*, 1990*a*). Affected patients not only have a high probability of developing RCC (70% at age 60 years), but also have an early age at onset and frequently develop multiple tumours (Maher *et al.*, 1990*a*, *b*). The gene for VHL disease has been mapped to chromosome 3p25-p26 (Seizinger *et al.*, 1988; Hosoe *et al.*, 1990; Maher *et al.*, 1991; Seizinger *et al.*, 1991*a*; Crossey *et al.*, 1993*a*; Richards *et al.*, 1993) and appears to function as a tumour-suppressor gene (Tory *et al.*, 1989; Maher *et al.*, 1990*b*; Crossey *et al.*, 1993*b*; Latif *et al.*, 1993). Another familial RCC gene (*RCC1*) also maps to the short arm of chromosome 3: Cohen *et al.* (1979) reported a large family in which a balanced translocation between chromosome 3 and 8 was associated with a predisposition to early-onset multicentric RCC. The translocation breakpoint was at chromosome 3p14, suggesting that mutations in two genes on chromsome 3p (*VHL* at 3p25-p26 and *RCC1* at 3p14) may cause familial RCC.

Mutations in one or more tumour-suppressor genes on chromosome 3p have also been implicated in the pathogenesis of non-familial RCC (Maher & Yates, 1991). Shimuzu *et al.* (1990) found that the effect of introducing a normal chromosome 3p into a RCC cell line was to suppress its tumorigenicity. In addition, cytogenetic and molecular studies of sporadic RCC have shown frequent chromosome 3p deletions (Zbar *et al.*, 1987; Kovacs *et al.*, 1988; Bergerheim *et al.*, 1989; Anglard *et al.*, 1991; Van der Hout *et al.*, 1991; Yamakawa *et al.*, 1991). The *VHL* and *RCC1* genes are candidate genes for non-familial RCC, but molecular genetic studies of chromosome 3p allele loss in sporadic RCC have yielded conflicting results about the localisation of the critical region of allele loss: Van der Hout *et al.* (1991) suggested 3p21, Yamakawa *et al.* (1991) suggested 3p14 and 3p21, and Bergerheim *et al.* (1989) and Anglard *et al.* (1991) suggested chromosome 3p21-p26, which would include the VHL disease locus. In addition to allele loss on chromosome 3p, loss of heterozygosity has been reported on several other chromosomes (including 5, 11 and 17) in sporadic RCC (Anglard *et al.*, 1991; Morita *et al.*, 1991). We have analysed non-familial RCC for allele loss in the region of known or putative tumour-suppressor genes on the short arm of chromosome 3 and on chromosomes 5, 11, 17 and 22 to investigate the molecular pathogenesis of sporadic RCC.

### Materials and methods

### Patient and tumour material

Paired blood-tumour samples ($n = 55$) from 55 patients (41 male, 14 female, mean age 56 years, range 22-77 years) with non-familial RCC were analysed for loss of heterozygosity at 14 loci located close to known or putative tumour-suppressor genes. All tumours samples were taken from primary tumours in previously untreated patients, and were snap frozen in liquid nitrogen and stored at $-30°C$ or $-70°C$ until analysed. All patients had a histologically proven diagnosis of RCC.

### Molecular genetic analysis and detection of allele loss

High molecular weight DNA was isolated from peripheral blood and frozen tumour tissue by standard methods (Sambrook *et al.*, 1989). Details of the loci investigated are given in Table I: eight loci mapped to chromosome 3p and six to other chromosomes. The locations of the chromosome 3p probes are given in Table I and in Figure 1. Three areas on chromsome 3p were of particular interest (see above): (i) chromosome 3p14 (D3S659 and D3S1067 flank the translocation breakpoint; Yamakawa *et al.*, 1992), (ii) chromosome 3p21-p24, a region that shows frequent LOH in a variety of tumour types, and (iii) chromosome 3p25-p26 close to the VHL disease tumour-suppressor gene (the locus order within this region is D3S651-D3S1038-D3S1317-VHL). The other loci were selected because they map close to tumour-suppressor genes: (i) *APC*/*MCC* genes at chromosome 5q21, (ii) *WT2* gene in chromosome 11p15.5, (iii) chromosome 17p (the p53 tumour-suppressor gene maps to 17p13.1 and we also investigated a marker at 17p13.2), (iv) chromsome 17q [the familial breast cancer gene (*BRCA1*) is located at 17q21], (v) the neurofibromatosis type 2 (*NF2*) gene on chromosome 22.

For the analysis of microsatellite markers (see Table I)

Correspondence: E.R. Maher, University of Cambridge, Box 134, Addenbrooke's Hospital, Hills Road, Cambridge CB2 2QQ, UK.
Received 18 May 1993; and in revised form 30 July 1993.

**Table I** Details of loci investigated for loss of heterozygosity

| *Locus* | *Location* | *Heterozygosity* | *Reference* |
|---|---|---|---|
| D3S659 | 3p13 | 0.73 | Jones *et al.* (1992) |
| D3S1228 | 3p14.1-14.3 | 0.77 | Jones *et al.* (1992) |
| D3S1076 | 3p21.1 | 0.59 | Jones *et al.* (1992) |
| D3S1067 | 3p14.3-p21.1 | 0.86 | Jones *et al.* (1992) |
| D3S647 | 3p23 | 0.73 | Jones *et al.* (1992) |
| D3S651 | 3p25 | 0.34 | Jones *et al.* (1992) |
| D3S1038 | 3p25 | 0.80 | Jones *et al.* (1992) |
| D3S1317 | 3p25-p26 | 0.70 | Tory *et al.* (1993) |
| D5S346 | 5q21 | 0.5 | Spirio *et al.* (1993) |
| D111S576 | 11p15.5 | 0.55 | Saito *et al.* (1992), Jones *et al.* (1993) |
| CI17-732CA | 17p13.2 | 0.60 | Jones *et al.* (1993) |
| p53 | 17p13.1 | 0.90 | Jones and Nakamura (1992) |
| D17S588 | 17q21 | 0.45 | S. Smith and B.A.J. Ponder (personal communication, 1993) |
| D22S268 | 22q12 | 0.71 | Marineau *et al.* (1993) |

**Figure 1** Localisation of chromosome 3p loci investigated.

DNA was amplified by the polymerase chain reaction (PCR) as described previously (Crossey *et al.*, 1993*a*, *b*). DNA (50 ng) was amplified by PCR in 20 µl reactions containing standard PCR buffer (10 mM Tris-Cl pH 8.8, 50 mM potassium chloride, 0.01% gelatin, 1.5 mM magnesium chloride, 10 pmol of each primer, 0.1 pmol of end-labelled primer, 200 µM each of dATP, dCTP, dGTP and dTTP, and 0.5 U of *Taq* polymerase. The samples were subjected to 20-30 PCR amplification cycles of 1 min denaturation at 94°C, 1 min annealing at 50-60°C and 1 min extension at 72°C. The PCR products were mixed with an equal volume of formamide loading buffer, heat denatured and then fractionated on a 6% polyacrylamide-6 M urea gel using a sequencing reaction as a size marker. Gels were dried and exposed for 1-3 days at -20°C.

### Results

### *Chromosome 3p*

All 55 tumours were informative at one or more loci on chromosome 3p, and overall 35 (64%) tumours showed LOH at one or more loci on chromosome 3p (see Table II). The 35 tumours with LOH on chromosome 3p could be divided into four groups according to the pattern of LOH: group a, 15 tumours showed LOH at all informative loci on chromosome 3p (tumours 6, 10, 11, 16, 17, 20, 23, 27, 29, 35, 36, 38, 42, 48, 54); group b, 15 tumours had LOH on chromosome 3p13-p24, but retention of heterozygosity in chromosome 3p25-p26 (tumours 4, 7, 12, 14, 15, 18, 19, 21, 22, 34, 37, 41, 44, 46, 53); group c, four tumours showed partial chromosome 3p allele loss including chromosome 3p25-p26 (1, 33, 40, 50); group d, tumour 9 showed a more complicated pattern with two non-contiguous regions of LOH. There were no significant correlations between chromosome 3p allele loss and sex or age at diagnosis. However, none of the four tumours classified as papillary RCC on histopathological examination (tumours 24, 39, 49 and 52) showed LOH on chromosome 3p, compared with 35 of 51 non-papillary RCC [$\chi^2$ (with Yates' correction) = 4.88, $P < 0.05$].

### *Other regions*

The results of loss of heterozygosity studies on chromosomes 5, 11, 13, 17 and 22 are shown in Table III. 1/46 (2%) informative tumours showed LOH at chromosome 5q21, 1/35 (3%) at chromosome 17p13.2, 5/39 (13%) at p53, 2/35 (6%) at chromosome 17q21 and 1/40 (3%) at D22S268 (see Figure 2). There was no relationship between the presence or absence of LOH at chromosome 3p and at other locations (4 of 35 tumours with chromosome 3p LOH had LOH and 3 of 20 with no chromosome 3p LOH had LOH at a non-chromosome 3 locus respectively; $\chi^2 = 0.15$, $P > 0.1$).

### Discussion

We have confirmed that chromosome 3p allele loss is the most frequent abnormality in sporadic RCC. Three candidate regions have been proposed to contain RCC tumour-suppressor genes (3p25-p26, 3p21 and 3p13-p14). The recent cloning of the VHL disease gene and the demonstration of inactivating mutations in five sporadic RCC cell lines has confirmed the hypothesis that VHL gene mutations are involved in the pathogenesis of sporadic RCC (Latif *et al.*, 1993). Each of the five RCC cell lines reported by Latif *et al.* (1993) contained a large chromosome 3p deletion (so that one VHL allele was lost) and a VHL gene mutation on the cytogenetically normal chromosome 3. Further studies to define the proportion of primary sporadic RCC with VHL gene mutations are in progress. Nevertheless, analysis of the pattern of allele loss in group B tumours suggests that other loci on the short arm of chromosome 3, in addition to the VHL gene, may be involved in the pathogenesis of sporadic RCC. Fifteen tumours showed chromosome 3p allele loss that did not involve the VHL region. Detailed analysis of the pattern of LOH in these tumours suggested two conclusions.

**Table II** Chromosome 3p allele loss in 55 sporadic RCCs

| | | | | | | | | | | | | | | *Tumour* | | | | | | | | | | | | | | |
|---|---|---|---|---|---|---|---|---|---|---|---|---|---|---|---|---|---|---|---|---|---|---|---|---|---|---|---|---|
| *Locus* | *6* | *10* | *11* | *16* | *17* | *20* | *23* | *27* | *29* | *35* | *36* | *38* | *42* | *48* | *54* | *4* | *7* | *12* | *14* | *15* | *18* | *19* | *21* | *22* | *34* | *37* | *41* | *44* | *46* |
| D3S1317 | ■ | – | ■ | ■ | ■ | ■ | – | ■ | – | – | – | ■ | – | ■ | – | – | □ | – | – | – | □ | – | □ | □ | □ | – | □ | – | □ |
| D3S1038 | ■ | ■ | ■ | ■ | ■ | – | ■ | ■ | ■ | ■ | ■ | ■ | ■ | ■ | ■ | □ | – | □ | – | □ | □ | – | □ | □ | ■ | – | □ | – | – |
| D3S1110 | ■ | – | ■ | ■ | ■ | ■ | ■ | – | ■ | – | ■ | ■ | ■ | ■ | – | – | – | – | – | – | – | □ | □ | – | – | □ | □ | – | – |
| D3S647 | ■ | ■ | ■ | – | – | – | – | ■ | – | – | – | – | – | ■ | – | – | – | □ | □ | □ | – | ■ | – | – | – | – | ■ | – | – |
| D3S1067 | ■ | ■ | – | – | ■ | ■ | ■ | – | – | ■ | ■ | – | ■ | ■ | – | □ | ■ | ■ | ■ | – | ■ | ■ | □ | ■ | ■ | □ | ■ | □ | □ |
| D3S1076 | – | – | – | ■ | – | ■ | – | ■ | ■ | – | ■ | ■ | ■ | – | ■ | – | – | ■ | ■ | ■ | – | – | – | – | □ | ■ | – | ■ | – |
| D3S1228 | ■ | ■ | – | ■ | ■ | – | – | ■ | – | – | – | ■ | – | ■ | – | ■ | □ | ■ | – | – | □ | – | ■ | – | – | – | – | ■ | – |
| D3S659 | ■ | ■ | ■ | ■ | ■ | ■ | – | ■ | ■ | – | ■ | ■ | ■ | ■ | ■ | ■ | – | ■ | – | – | □ | – | □ | □ | – | – | ■ | ■ | ■ |

| | | | | | | | | | | | | | *Tumour* | | | | | | | | | | | | | |
|---|---|---|---|---|---|---|---|---|---|---|---|---|---|---|---|---|---|---|---|---|---|---|---|---|---|---|
| *Locus* | *53* | *1* | *33* | *40* | *50* | *9* | *2* | *3* | *5* | *8* | *13* | *24* | *25* | *26* | *28* | *30* | *31* | *32* | *39* | *43* | *45* | *47* | *49* | *51* | *52* | *55* |
| D3S1317 | □ | – | – | ■ | – | ■ | – | □ | – | □ | □ | □ | – | □ | – | □ | □ | □ | □ | □ | □ | □ | – | □ | – | – |
| D3S1038 | ■ | – | ■ | ■ | ■ | □ | – | □ | □ | – | – | □ | □ | □ | – | □ | □ | □ | □ | □ | – | □ | – | □ | □ | – |
| D3S651 | – | – | – | – | – | – | □ | □ | – | – | – | □ | □ | – | □ | □ | – | – | □ | – | – | □ | – | □ | – | – |
| D3S647 | – | ■ | – | □ | □ | – | □ | – | – | □ | □ | □ | – | – | – | – | □ | □ | – | – | – | □ | □ | – | □ | – |
| D3S1067 | ■ | □ | ■ | – | – | ■ | □ | □ | □ | – | – | □ | □ | □ | □ | □ | – | □ | □ | □ | □ | □ | □ | □ | – | – |
| D3S1076 | – | – | – | □ | □ | – | – | – | □ | □ | – | – | – | – | – | – | – | □ | □ | – | – | □ | □ | – | – | – |
| D3S1228 | ■ | – | □ | – | – | □ | □ | □ | – | – | □ | □ | – | □ | – | – | – | – | □ | – | □ | □ | □ | □ | – | – |
| D3S659 | – | – | – | □ | □ | – | □ | □ | – | – | □ | □ | □ | □ | □ | □ | – | □ | □ | □ | □ | □ | □ | □ | □ | □ |

Key: ■ = loss of heterozygosity, □ = retention of heterozygosity; – = uninformative or failed.

**Table III** Molecular genetic analysis of 55 sporadic RCCs: loss of heterozygosity studies on chromosomes 5, 11, 17 and 22

| | | | | | | | | | | | | | | *Tumour* | | | | | | | | | | | | | | |
|---|---|---|---|---|---|---|---|---|---|---|---|---|---|---|---|---|---|---|---|---|---|---|---|---|---|---|---|---|
| *Locus* | *6* | *10* | *11* | *16* | *17* | *20* | *23* | *27* | *29* | *35* | *36* | *38* | *42* | *48* | *54* | *4* | *7* | *12* | *14* | *15* | *18* | *19* | *21* | *22* | *34* | *37* | *41* | *44* | *46* |
| D3S13346 | □ | – | □ | □ | □ | □ | □ | □ | □ | □ | □ | □ | □ | □ | □ | □ | □ | □ | □ | □ | □ | □ | □ | □ | □ | □ | □ | □ | – |
| D11S576 | □ | – | □ | □ | – | – | – | □ | □ | – | □ | – | – | □ | – | □ | – | – | □ | □ | – | – | – | – | □ | – | □ | □ | – |
| CI17-732 | – | □ | – | □ | – | □ | – | □ | – | □ | – | □ | □ | □ | □ | □ | – | □ | – | – | □ | □ | □ | – | □ | – | □ | □ | – |
| p53 | □ | □ | – | □ | □ | □ | □ | □ | – | – | ■ | ■ | – | □ | □ | □ | □ | □ | □ | – | □ | □ | □ | – | □ | □ | □ | – | □ |
| D17S588 | □ | □ | □ | □ | □ | – | □ | □ | – | □ | ■ | – | □ | □ | □ | □ | – | □ | – | □ | □ | – | □ | – | □ | □ | – | □ | □ |
| D22S268 | □ | □ | – | □ | □ | □ | – | □ | □ | □ | – | – | □ | – | □ | □ | □ | ■ | – | □ | □ | □ | □ | □ | □ | □ | – | □ | □ |

| | | | | | | | | | | | | | *Tumour* | | | | | | | | | | | | | |
|---|---|---|---|---|---|---|---|---|---|---|---|---|---|---|---|---|---|---|---|---|---|---|---|---|---|---|
| *Locus* | *53* | *1* | *33* | *40* | *50* | *9* | *2* | *3* | *5* | *8* | *13* | *24* | *25* | *26* | *28* | *30* | *31* | *32* | *39* | *43* | *45* | *47* | *49* | *51* | *52* | *55* |
| D3S346 | □ | □ | □ | □ | □ | □ | □ | – | – | – | □ | – | □ | □ | □ | □ | □ | ■ | – | – | – | □ | □ | □ | □ | □ |
| D11S576 | – | □ | ■ | □ | – | □ | □ | □ | □ | □ | – | – | □ | □ | □ | – | – | – | □ | – | – | – | – | – | – | □ |
| CI17-732 | – | □ | □ | □ | □ | □ | □ | □ | – | □ | – | □ | □ | □ | □ | – | □ | ■ | □ | □ | – | □ | – | – | □ | – |
| p53 | – | □ | – | □ | □ | □ | □ | □ | □ | – | □ | □ | □ | □ | □ | ■ | – | ■ | – | – | – | □ | – | □ | □ | ■ |
| D17S588 | – | – | – | □ | – | □ | – | □ | □ | □ | – | □ | □ | □ | □ | – | □ | ■ | □ | – | – | □ | – | – | □ | – |
| D22S268 | – | □ | □ | □ | □ | – | – | □ | □ | □ | □ | – | – | □ | □ | □ | □ | – | – | □ | □ | □ | – | □ | □ | – |

Key: ■ = loss of heterozygosity, □ = retention of heterozygosity; – = uninformative or failed.

**Figure 2** Examples of loss of heterozygosity at loci on chromosome 3p (tumour 36) and 17 (tumours 36 and 32 respectively). Key: B = blood DNA; T = tumour DNA.

Firstly, if a single tumour-suppressor gene was involved it should be located centromeric to D3S1067 (see tumours 4, 21, 37, 44 and 46) and telomeric to D3S1076 (see tumour 34). However this conclusion is dependent on the deletion in tumour 34 overlapping with that in tumour 4, 21, 37, 44 or 46, and this could not be determined because DNA markers mapping between D3S1067 and D3S1076 were not available for study. The alternative conclusion would be that if the deletion in tumour 34 and those in tumours 4, 21, 37, 44 or 46 did not overlap then two tumour-suppressor genes, at 3p14 and 3p21, might be involved, as suggested by Yamakawa *et al.* (1991). Following the isolation of the *RCC1* gene it will be possible to investigate the role of *RCC1* mutations in the pathogenesis of sporadic RCC. In addition, the isolation and accurate mapping of more microsatellite markers from chromosome 3p14–p21 would enable the critical region of chromosome 3p allele loss in sporadic RCC to be defined more precisely.

Human carcinogenesis is characteristically a multistep process in which mutations accumulate in a restricted number of tumour-suppressor genes and oncogenes. Although chromosome 3p allele loss is a frequent event in sporadic RCC, mutations in other tumour-suppressor genes may also occur. Morita *et al.* (1991) reported chromosome 17p and 5q allele loss in 5/24 and 5/17 RCCs respectively. However, Horii *et al.* (1992) did not detect any *APC* gene mutations in RCC with chromosome 5q LOH and suggested that another tumour-suppressor gene on chromosome 5q might contribute to the pathogenesis of RCC. The lower rates of LOH at chromosome 5q21 and 22q found by us (1/46 and 1/40 informative tumours respectively) are similar to those reported by van der Hout *et al.* (1991) (0/9 and 0/8 respectively). We detected LOH on chromosome 17 most frequently in the region of the p53 tumour-suppressor gene, although most tumours with LOH at p53 also demonstrated LOH at other chromosome 17 loci investigated. Although p53 mutations are the most frequent genetic abnormality in human cancer, the frequency of p53 involvement in sporadic RCC is less than in many other tumour types. We found LOH at the p53 locus in only 13% of informative tumours, which is similar to the findings of van der Hout *et al.* (1991) (12.5% LOH on 17p), Anglard *et al.* (1991) (11% LOH on chromosome 17), Torigoe *et al.* (1992) (10% p53 mutations), Whaley *et al.* (1990) (7% p53 mutations) and Suzuki *et al.* (1992) (4.3% p53 mutations). It has been suggested that chromosome 3p allele loss is an early event in RCC, but that other tumour-suppressor gene mutations are involved in tumour progression. Anglard *et al.* (1991) found that LOH at chromosome 11p and 13 was not present in localised tumours but was frequent in stage IV tumours. Kovacs *et al.* (1989) related the histopathological features of non-familial RCC with the molecular pathology, and suggested that chromosome 3p allele loss is infrequent in the papillary subgroup of RCC. Our findings also support this association.

The isolation of hereditary cancer genes will allow their role in the pathogenesis of non-familial RCC to be investigated by direct mutation analysis. Such studies should also elucidate the relationship between RCC tumour-suppressor genes and the molecular pathology of other human cancers, such as lung, breast, ovary, uterus and testis cancer, which show frequent chromosome 3p allele loss (Seizinger *et al.*, 1991*b*).

We thank the Cancer Research Campaign, Action Research and the National Kidney Research Fund for financial support and the many colleagues who helped in the collection of tumour samples.

### References

ANGLARD, P., TORY, K., BRAUCH, H., WEISS, G.H., LATIF, F., MERINO, M.J., LERMAN, M.I., ZBAR, B. & LINEHAN, W.M. (1991). Molecular analysis of genetic changes in the origin and development of renal cell carcinoma. *Cancer Res.*, **51**, 1071–1077.

BERGERHEIM, U., NORDENSKJÖLD, M. & COLLINS, V.P. (1989). Deletion mapping in human renal cell carcinoma. *Cancer Res.*, **49**, 1390–1396.

COHEN, A.J., LI, F.P., BERG, S., MARCHETTO, D.J., TSAI, S., JACOBS, S.C. & BROWN, R.S. (1979). Hereditary renal cell carcinoma associated with a chromosomal translocation. *N. Engl. J. Med.*, **301**, 592–595.

CROSSEY, P.A., MAHER, E.R., JONES, M.H., RICHARDS, F.M., LATIF, F., PHIPPS, M.E., LUSH, M., FOSTER, K., TORY, K., GREEN, J.S., OOSTRA, B., YATES, J.R.W., LINEHAN, W.M., AFFARA, N.A., LERMAN, M., ZBAR, B., NAKAMURA, Y. & FERGUSON-SMITH, M.A. (1993*a*). Genetic linkage between von Hippel-Lindau disease and three microsatellite polymorphisms refines the localisation of the VHL locus. *Hum. Mol. Genet.*, **2**, 279-282.

CROSSEY, P.A., MAHER, E.R., FOSTER, K., RICHARDS, F.M., LATIF, F., TORY, K., JONES, M.H., BENTLEY, E., LERMAN, M.I., ZBAR, B., AFFARA, N.A. & FERGUSON-SMITH, M.A. (1993*b*). Molecular genetic investigation of the mechanism of tumourigenesis in von Hippel-Lindau disease: analysis of allele loss in VHL tumours. *Hum. Genet.* (in press).

HORII, A., NAKATSURU, S., MIYOSHI, Y., ICHII, S., NAGASE, H., ANDO, H., YANAGISAWA, A., TSUCHIYA, E., KATO, Y. & NAKAMURA, Y. (1992). Frequent somatic mutations of the APC gene in human pancreatic cancer. *Cancer Res.*, **52**, 6696-6698.

HOSOE, S., BRAUCH, H., LATIF, F., GLENN, G., DANIEL, L., BALE, S., CHOYKE, P., GORIN, M., OLDFIELD, E., BERMAN, A., GOODMAN, J., ORCUTT, M.L., HAMPSCH, K., DELISIO, J., MODI, W., MCBRIDE, W., ANGLARD, P., WEISS, G., WALTHER, M.J., LINEHAN, W.M., LERMAN, M.I. & ZBAR, B. (1990). Localization of the von Hippel-Lindau disease gene to a small region of chromosome 3. *Genomics*, **8**, 634-640.

JONES, M.H. & NAKAMURA, Y. (1992). Detection of loss of heterozygosity at the human TP53 locus using a dinucleotide repeat. *Genes Chrom. Cancer*, **5**, 89-90.

JONES, M.H., YAMAKAWA, K. & NAKAMURA, Y. (1992). Isolation and characterisation of 19 dinucleotide repeat polymorphisms on chromosome 3p. *Hum. Mol. Genet.*, **1**, 131-133.

JONES, M.H., SATO, T., SAITO, H., TANIGAMI, A. & NAKAMURA, Y. (1993). Microsatellite polymorphisms at candidate and confirmed tumour suppressor gene loci for linkage and loss of heterozygosity analysis (in preparation).

KOVACS, G., ERLANDSSEN, R., BOLDOG, F., INGVARSSON, S., MULLER-BRECLIN, R., KLEIN, G. & SUMEGI, J. (1988). Consistent chromosome 3p deletion and loss of heterozygosity in renal cell carcinoma. *Proc. Natl Acad. Sci. USA*, **85**, 1571-1575.

KOVACS, G., WILKENS, L., PAPP, T. & DE RIESE, W. (1989). Differentiation between papillary and nonpapillary renal cell carcinomas by DNA analysis. *J. Natl Cancer Inst.*, **81**, 527-530.

LATIF, F., TORY, K., GNARRA, J., YAO, M., DUH, F.-M., ORCUTT, M.L., STACKHOUSE, T., KUZMIN, I., MODI, W., GEIL, L., SCHMIDT, L., ZHOU, F., LI, H., WEI, M.H., CHEN, F., GLENN, G., CHOYKE, P., WALTHER, M.M., WENG, Y., DUAN, D.R., DEAN, M., GLAVAC, D., RICHARDS, F.M., CROSSEY, P.A., FERGUSON-SMITH, M.A., LE PASLIER, D., CHUMAKOV, I., COHEN, D., CHINAULT, C.A., MAHER, E.R., LINEHAN, W.M., ZBAR, B. & LERMAN, M.I. (1993). Isolation of the von Hippel-Lindau disease tumour suppressor gene. *Science*, **260**, 1317-1320.

MAHER, E.R. & YATES, J.R.W. (1991). Familial renal cell carcinoma: clinical and molecular genetic aspects (editorial). *Br. J. Cancer*, **63**, 176-179.

MAHER, E.R., YATES, J.R.W., HARRIES, R., BENJAMIN, C., HARRIS, R., MOORE, A.T. & FERGUSON-SMITH, M.A. (1990*a*). Clinical features and natural history of von Hippel-Lindau disease. *Q. J. Med.*, **77**, 1151-1163.

MAHER, E.R., YATES, J.R.W. & FERGUSON-SMITH, M.A. (1990*b*). Statistical analysis of the two stage mutation model in von Hippel-Lindau disease, and in sporadic cerebellar haemangioblastoma and renal cell carcinoma. *J. Med. Gen.*, **27**, 311-314.

MAHER, E.R., BENTLEY, E., YATES, J.R.W., LATIF, F., LERMAN, M., ZBAR, B., AFFARA, N.A., FERGUSON-SMITH, M.A. (1991). Mapping of the von Hippel-Lindau disease locus to a small region of chromosome 3p by genetic linkage analysis. *Genomics*, **10**, 957-960.

MARINEAU, C., BARON, C., DELATTRE, O., ZUCMAN, J., THOMAS, G. & ROULEAU, G.A. (1993). Dinucleotide repeat polymorphism at the D22S268 locus. *Hum. Mol. Genet.*, **2**, 336.

MORITA, R., ISHIKAWA, J., TSUTSUMI, M., HIKIJI, K., TUSUKADA, Y., KAMIDONO, S., MAEDA, S. & NAKAMURA, Y. (1991). Allelotype of renal cell carcinoma. *Cancer Res.*, **51**, 820-823.

RICHARDS, F.M., MAHER, E.R., LATIF, F., PHIPPS, M.E., TORY, K., LUSH, M., CROSSEY, P.A., OOSTRA, B., GUSTAVSON, K.H., GREEN, J., TURNER, G., YATES, J.R.W., LINEHAN, W.M., AFFARA, N.A., LERMAN, M., ZBAR, B. & FERGUSON-SMITH, M.A. (1993). Detailed genetic mapping of the von Hippel-Lindau disease tumour suppressor gene. *J. Med. Genet.*, **30**, 104-107.

SAITO, S., OKUI, K., TOKINO, T., OSHIMURA, M. & NAKAMURA, Y. (1992). Isolation and mapping of 68 RFLP markers on human chromosome 6. *Am. J. Hum. Genet.*, **50**, 65-70.

SAMBROOK, J., FRITSCH, E.F. & MANIATIS, T. (1989). *Molecular Cloning: A Laboratory Manual.* Cold Spring Harbor Laboratory Press: Cold Spring Harbor, NY.

SEIZINGER, B.R., ROULEAU, G.A., OZELIUS, L.J., LANE, A.H., FARMER, G.E., LAMIELL, J.M., HAINES, J., YUEN, J.W., COLLINS, D., MAJOOR-KRAKAUER, D., BONNER, T., MATHEW, C., RUBENSTEIN, A., HALPERIN, J., MCCONKIE-ROSELL, A., GREEN, J.S., TRPFATTER, J.A., PONDER, B.A., EIERMAN, L., BOWMER, M.I., SCHIMKE, R., OOSTRA, B., ARONIN, N., SMITH, D.I., DRABKIN, H., WAZIRI, M.H., HOBBS, W.J., MARTUZA, R.L., CONNEALLY, P.M., HSIA, Y.E. & GUSELLA, J.F. (1988). Von Hippel-Lindau disease maps to the region of chromosome 3 associated with renal cell carcinoma. *Nature*, **332**, 268-269.

SEIZINGER, B.R., SMITH, D.I., FILLING-KATZ, M.R., NEUMANN, H., GREEN, J.S., CHOYKE, P.L., ANDERSON, K.M., FREIMAN, R.N., HSIA, Y.E., COLLINS, D., HALPERIN, J., LAMIELL, J.M., OOSTRA, B., WAZIRI, M.H., GORIN, M.B., SCHERER, G., DRABKIN, H.A., ARONIN, N., SCHINZEL, A., MARTUZA, R.L., GUSELLA, J.F. & HAINES, J.L. (1991*a*). Genetic flanking markers refine diagnostic criteria and provide insights into the genetics of Von Hippel-Lindau disease. *Proc. Natl Acad. Sci. USA*, **88**, 2864-2868.

SEIZINGER, B.R., KLINGER, H.P., JUNIEN, C., NAKAMURA, Y., LE BEAU, M., CAVANEE, W., EMANUEL, B., PONDER, B., NAYLOR, S., MITELMAN, F., LOUIS, D., MENON, A., NEWSHAM, I., DECKER, J., KAELBLING, M., HENRY, I. & DEIMLING, A.V. (1991*b*). Report of the committee on chromosome and gene loss in human neoplasia. *Cytogenet. Cell Genet.*, **58**, 1080-1096.

SHIMIZU, M., YOKOTA, J., MORI, N., SHUIN, T., SHINODA, M., TERADA, M. & OSHIMURA, M. (1990). Introduction of normal chromosome 3p modulates the tumorigenicity of a human renal cell carcinoma cell line YCR. *Oncogene*, **5**, 185-194.

SPIRIO, L., NELSON, L., WARD, K., BURT, R., WHITE, R. & LEPPERT, M. (1993). A CA-repeat polymorphism close to the adenomatous polyposis coli (APC) gene offers improved diagnostic testing for familial APC. *Am. J. Hum. Genet.*, **52**, 286-296.

SUZUKI, Y., TAMURA, G., SATODATE, R. & FUJIOKA, T. (1992). Infrequent mutation of p53 gene in human renal cell carcinoma detected by polymerase chain reaction single-strand conformation polymorphism analysis. *Jpn. J. Cancer Res.*, **83**, 233-235.

TORIGOE, S., SHUIN, T., KUBOTA, Y., HORIKOSHI, T., DANENBERG, K. & DANENBERG, P.V. (1992). p53 gene mutation in primary human renal cell carcinoma. *Oncology Res.*, **4**, 467-472.

TORY, K., BRAUCH, H., LINEHAN, M., BARBA, D., OLDFIELD, E., FILLING-KATZ, M., SEIZINGER, B., NAKAMURA, Y., WHITE, R., MARSHALL, F.F., LERMAN, M.I. & ZBAR, B. (1989). Specific genetic change in tumors associated with von Hippel-Lindau disease. *J. Natl Cancer Ins.*, **81**, 1097-1101.

TORY, K., LATIF, F., MODI, W., SCHMIDT, L., WEI, M.H., LI, H., COBLER, P., ORCUTT, M.L., DELISIO, J., GEIL, L., ZBAR, B. & LERMAN, M.I. (1992). A genetic linkage map of 96 loci on the short arm of human chromosome 3. *Genomics*, **13**, 275-286.

VAN DER HOUT, A.H., VAN DER VLIES, P., WIJMENGA, C., LI, F.P., OOSTERHUIS, J.W. & BUYS, C.H. (1991). The region of common allelic losses in sporadic renal cell carcinoma is bordered by the loci D3S2 and THRB. *Genomics*, **11**, 537-542

WHALEY, J.M., CHUNG, R.Y., YANDELL, D.W., MENON, A., LI, F.P. & SEIZINGER, B.R. (1990). Mutation of the p53 gene is an uncommon event in sporadic human renal cell carcinomas. *Am. J. Hum. Genet.*, **47**, A24.

YAMAKAWA, K., MORITA, R., TAKAHASHI, E., HORI, T., ISHIKAWA, J. & NAKAMURA, Y. (1991). A detailed deletion mapping of the short arm of chromosome 3 in sporadic renal cell carcinoma. *Cancer Res.*, **51**, 4707-4711.

YAMAKAWA, K., TAKAHASHI, E., MURATA, M., OKUI, K., YOKOYAMA, S. & NAKAMURA, Y. (1992). Detailed mapping around the breakpoint of (3;8) translocation in familial renal cell carcinoma and FRA3B. *Genomics*, **14**, 412-416.

ZBAR, B., BRAUCH, H., TALMADGE, C. & LINEHAN, M. (1987). Loss of alleles on loci on the short arm of chromosome 3 in renal cell carcinoma. *Nature*, **305**, 721-724.


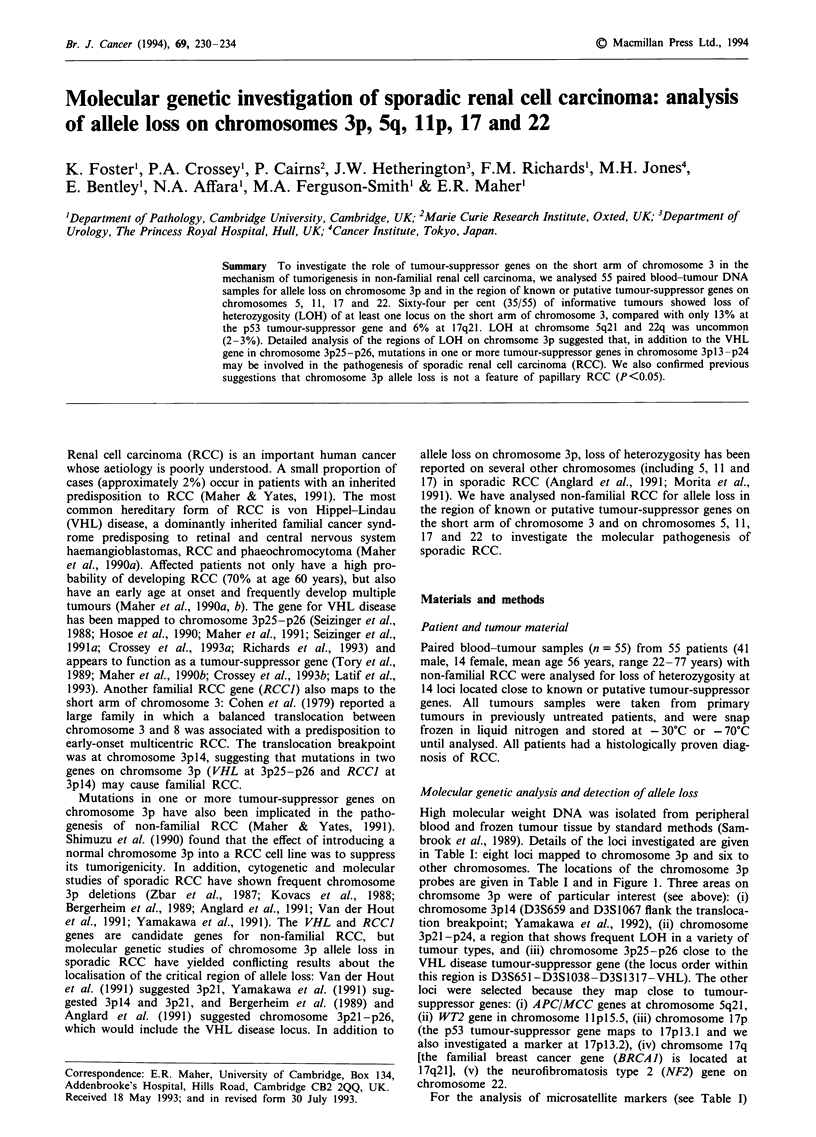

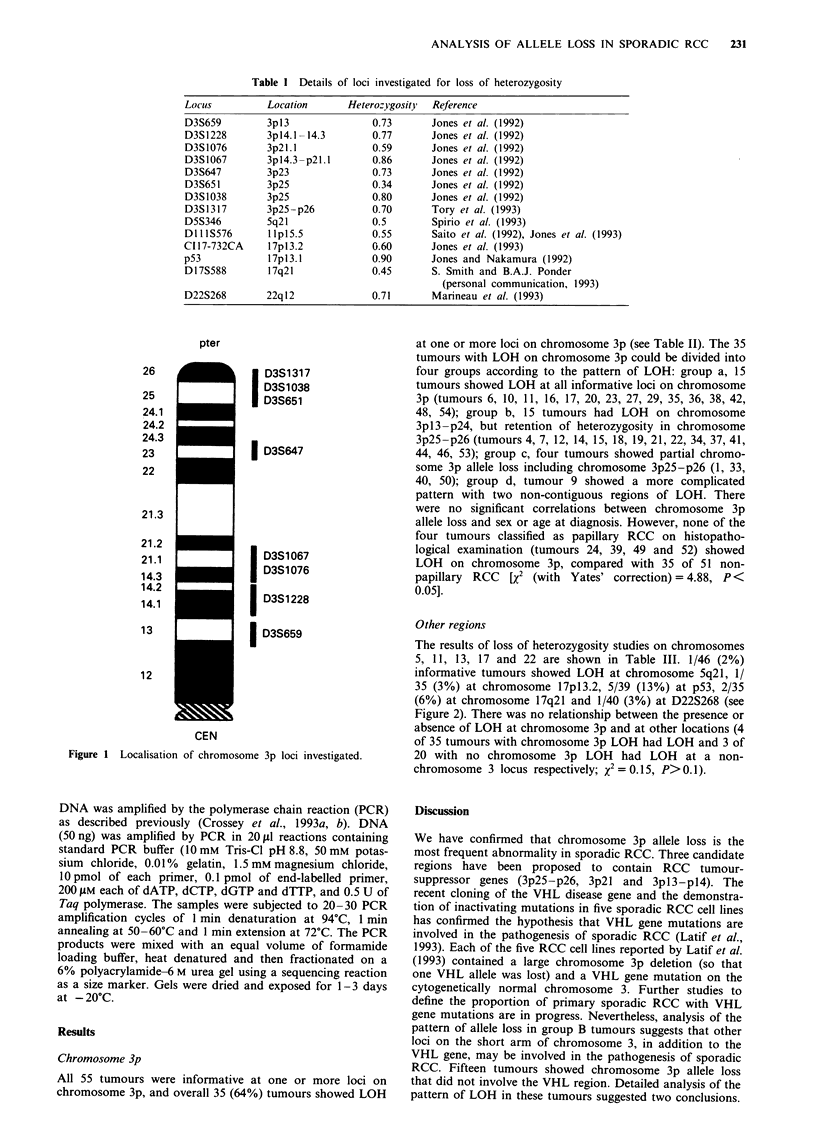

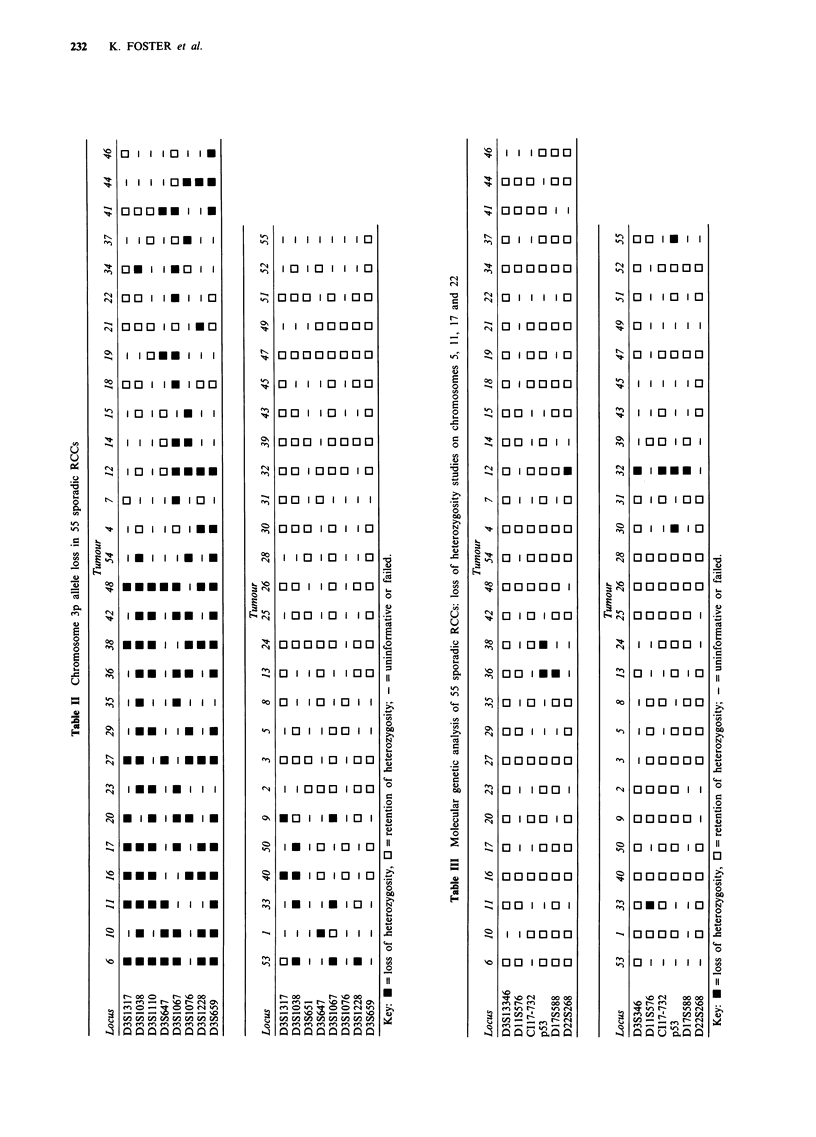

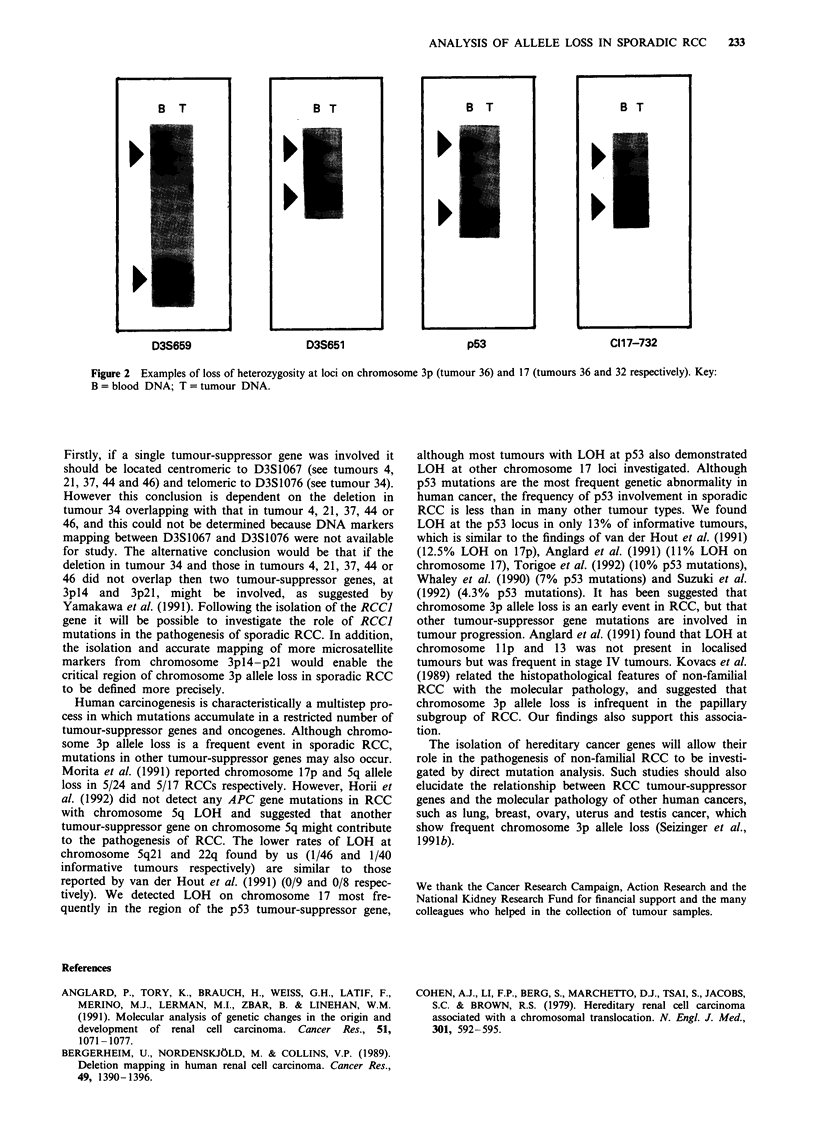

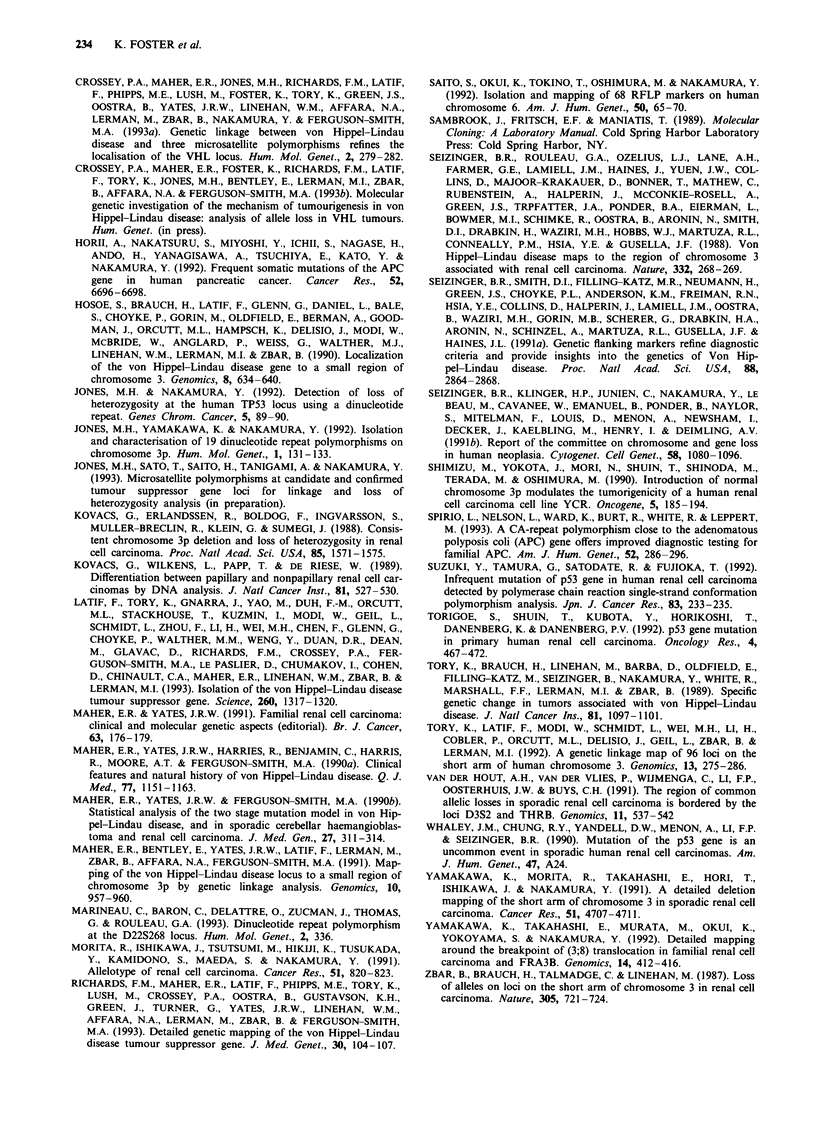


## References

[OCR_00849] Anglard P., Tory K., Brauch H., Weiss G. H., Latif F., Merino M. J., Lerman M. I., Zbar B., Linehan W. M. (1991). Molecular analysis of genetic changes in the origin and development of renal cell carcinoma.. Cancer Res.

[OCR_00856] Bergerheim U., Nordenskjöld M., Collins V. P. (1989). Deletion mapping in human renal cell carcinoma.. Cancer Res.

[OCR_00861] Cohen A. J., Li F. P., Berg S., Marchetto D. J., Tsai S., Jacobs S. C., Brown R. S. (1979). Hereditary renal-cell carcinoma associated with a chromosomal translocation.. N Engl J Med.

[OCR_00869] Crossey P. A., Maher E. R., Jones M. H., Richards F. M., Latif F., Phipps M. E., Lush M., Foster K., Tory K., Green J. S. (1993). Genetic linkage between von Hippel-Lindau disease and three microsatellite polymorphisms refines the localisation of the VHL locus.. Hum Mol Genet.

[OCR_00885] Horii A., Nakatsuru S., Miyoshi Y., Ichii S., Nagase H., Ando H., Yanagisawa A., Tsuchiya E., Kato Y., Nakamura Y. (1992). Frequent somatic mutations of the APC gene in human pancreatic cancer.. Cancer Res.

[OCR_00895] Hosoe S., Brauch H., Latif F., Glenn G., Daniel L., Bale S., Choyke P., Gorin M., Oldfield E., Berman A. (1990). Localization of the von Hippel-Lindau disease gene to a small region of chromosome 3.. Genomics.

[OCR_00901] Jones M. H., Nakamura Y. (1992). Detection of loss of heterozygosity at the human TP53 locus using a dinucleotide repeat polymorphism.. Genes Chromosomes Cancer.

[OCR_00906] Jones M. H., Yamakawa K., Nakamura Y. (1992). Isolation and characterization of 19 dinucleotide repeat polymorphisms on chromosome 3p.. Hum Mol Genet.

[OCR_00917] Kovacs G., Erlandsson R., Boldog F., Ingvarsson S., Müller-Brechlin R., Klein G., Sümegi J. (1988). Consistent chromosome 3p deletion and loss of heterozygosity in renal cell carcinoma.. Proc Natl Acad Sci U S A.

[OCR_00923] Kovacs G., Wilkens L., Papp T., de Riese W. (1989). Differentiation between papillary and nonpapillary renal cell carcinomas by DNA analysis.. J Natl Cancer Inst.

[OCR_00934] Latif F., Tory K., Gnarra J., Yao M., Duh F. M., Orcutt M. L., Stackhouse T., Kuzmin I., Modi W., Geil L. (1993). Identification of the von Hippel-Lindau disease tumor suppressor gene.. Science.

[OCR_00956] Maher E. R., Bentley E., Yates J. R., Latif F., Lerman M., Zbar B., Affara N. A., Ferguson-Smith M. A. (1991). Mapping of the von Hippel-Lindau disease locus to a small region of chromosome 3p by genetic linkage analysis.. Genomics.

[OCR_00939] Maher E. R., Yates J. R. (1991). Familial renal cell carcinoma: clinical and molecular genetic aspects.. Br J Cancer.

[OCR_00950] Maher E. R., Yates J. R., Ferguson-Smith M. A. (1990). Statistical analysis of the two stage mutation model in von Hippel-Lindau disease, and in sporadic cerebellar haemangioblastoma and renal cell carcinoma.. J Med Genet.

[OCR_00944] Maher E. R., Yates J. R., Harries R., Benjamin C., Harris R., Moore A. T., Ferguson-Smith M. A. (1990). Clinical features and natural history of von Hippel-Lindau disease.. Q J Med.

[OCR_00963] Marineau C., Baron C., Delattre O., Zucman J., Thomas G., Rouleau G. A. (1993). Dinucleotide repeat polymorphism at the D22S268 locus.. Hum Mol Genet.

[OCR_00968] Morita R., Ishikawa J., Tsutsumi M., Hikiji K., Tsukada Y., Kamidono S., Maeda S., Nakamura Y. (1991). Allelotype of renal cell carcinoma.. Cancer Res.

[OCR_00973] Richards F. M., Maher E. R., Latif F., Phipps M. E., Tory K., Lush M., Crossey P. A., Oostra B., Enblad P., Gustavson K. H. (1993). Detailed genetic mapping of the von Hippel-Lindau disease tumour suppressor gene.. J Med Genet.

[OCR_00981] Saito S., Okui K., Tokino T., Oshimura M., Nakamura Y. (1992). Isolation and mapping of 68 RFLP markers on human chromosome 6.. Am J Hum Genet.

[OCR_00994] Seizinger B. R., Rouleau G. A., Ozelius L. J., Lane A. H., Farmer G. E., Lamiell J. M., Haines J., Yuen J. W., Collins D., Majoor-Krakauer D. (1988). Von Hippel-Lindau disease maps to the region of chromosome 3 associated with renal cell carcinoma.. Nature.

[OCR_01003] Seizinger B. R., Smith D. I., Filling-Katz M. R., Neumann H., Green J. S., Choyke P. L., Anderson K. M., Freiman R. N., Klauck S. M., Whaley J. (1991). Genetic flanking markers refine diagnostic criteria and provide insights into the genetics of Von Hippel Lindau disease.. Proc Natl Acad Sci U S A.

[OCR_01022] Shimizu M., Yokota J., Mori N., Shuin T., Shinoda M., Terada M., Oshimura M. (1990). Introduction of normal chromosome 3p modulates the tumorigenicity of a human renal cell carcinoma cell line YCR.. Oncogene.

[OCR_01028] Spirio L., Nelson L., Ward K., Burt R., White R., Leppert M. (1993). A CA-repeat polymorphism close to the adenomatous polyposis coli (APC) gene offers improved diagnostic testing for familial APC.. Am J Hum Genet.

[OCR_01034] Suzuki Y., Tamura G., Satodate R., Fujioka T. (1992). Infrequent mutation of p53 gene in human renal cell carcinoma detected by polymerase chain reaction single-strand conformation polymorphism analysis.. Jpn J Cancer Res.

[OCR_01040] Torigoe S., Shuin T., Kubota Y., Horikoshi T., Danenberg K., Danenberg P. V. (1992). p53 gene mutation in primary human renal cell carcinoma.. Oncol Res.

[OCR_01046] Tory K., Brauch H., Linehan M., Barba D., Oldfield E., Filling-Katz M., Seizinger B., Nakamura Y., White R., Marshall F. F. (1989). Specific genetic change in tumors associated with von Hippel-Lindau disease.. J Natl Cancer Inst.

[OCR_01053] Tory K., Latif F., Modi W., Schmidt L., Wei M. H., Li H., Cobler P., Orcutt M. L., Delisio J., Geil L. (1992). A genetic linkage map of 96 loci on the short arm of human chromosome 3.. Genomics.

[OCR_01071] Yamakawa K., Morita R., Takahashi E., Hori T., Ishikawa J., Nakamura Y. (1991). A detailed deletion mapping of the short arm of chromosome 3 in sporadic renal cell carcinoma.. Cancer Res.

[OCR_01077] Yamakawa K., Takahashi E., Murata M., Okui K., Yokoyama S., Nakamura Y. (1992). Detailed mapping around the breakpoint of (3;8) translocation in familial renal cell carcinoma and FRA3B.. Genomics.

[OCR_01083] Zbar B., Brauch H., Talmadge C., Linehan M. Loss of alleles of loci on the short arm of chromosome 3 in renal cell carcinoma.. Nature.

[OCR_01059] van der Hout A. H., van der Vlies P., Wijmenga C., Li F. P., Oosterhuis J. W., Buys C. H. (1991). The region of common allelic losses in sporadic renal cell carcinoma is bordered by the loci D3S2 and THRB.. Genomics.

